# Increased Renal Versican Expression Is Associated with Progression of Chronic Kidney Disease

**DOI:** 10.1371/journal.pone.0044891

**Published:** 2012-09-14

**Authors:** Michael Rudnicki, Paul Perco, Hannes Neuwirt, Susie-Jane Noppert, Johannes Leierer, Judith Sunzenauer, Susanne Eder, Carlamaria Zoja, Kathrin Eller, Alexander R. Rosenkranz, Gerhard A. Müller, Bernd Mayer, Gert Mayer

**Affiliations:** 1 Medical University Innsbruck, Deptartment of Internal Medicine IV - Nephrology and Hypertension, Innsbruck, Austria; 2 Emergentec Biodevelopment GmbH, Vienna, Austria; 3 Mario Negri Institute for Pharmacological Research, Centro Anna Maria Astori, Science and Technology Park Kilometro Rosso, Bergamo, Italy; 4 Medical University of Graz, Deptartment of Internal Medicine, Clinical Division of Nephrology and Hemodialysis, Graz, Austria; 5 Georg-August-University Göttingen, Department of Medicine, Nephrology and Rheumatology, Göttingen, Germany; Inserm, France

## Abstract

Novel prognostic markers for progression of kidney disease are needed to distinguish patients who might benefit from a more aggressive nephroprotective therapy. Expression of the proteoglycan versican was evaluated in renal transcriptomics profiles and in an independent set of 74 renal biopsies. Versican levels were correlated to histologic damage scores and to renal outcome, and versican expression and regulation was evaluated *in vitro*. In transcriptomics profiles of renal tissue versican was positively correlated with (i) histological parameters in kidney biopsies, (ii) progressive decline of renal function in proteinuric kidney diseases, and (iii) impaired renal function and histology scores in diabetic nephropathy. In an independent cohort of 74 biopsies of glomerular diseases renal RNA levels of versican isoforms V0 and V1, but not V2 and V3 correlated significantly with creatinine after a mean follow up time of 53 months. Versican isoforms V0 and V1 together with serum creatinine at time of biopsy and the degree of glomerulosclerosis predicted 20% and 24% of the variability of creatinine at follow up, which was significantly more than serum creatinine and histological parameters alone (16%). However, when patients with acute kidney failure at time of biopsy (n = 5) were excluded, the additive predictive value of versican V1 was only marginally higher (35%) than creatinine and glomerulosclerosis alone (34%). Versican isoforms V0 and V1 were primarily expressed *in vitro* in proximal tubule cells and in fibroblasts. The results in humans were confirmed in three rodent models of kidney disease, in which renal versican expression was significantly upregulated as compared to corresponding controls. These data show for the first time an association of renal versican isoform V0 and V1 expression with progressive renal disease.

## Introduction

Progression of chronic kidney disease (CKD) is associated with increased morbidity and mortality, reduced quality of life, and major challenges for healthcare systems [1]. Clinical features predicting a poor prognosis include impaired renal function, hypertension and nephrotic range proteinuria at presentation [2] as well as during follow-up [3], [4]. Histopathological changes such as the degree of tubular atrophy and interstitial fibrosis have been shown to better predict long-term renal survival than the extent of glomerular damage even in primary glomerular diseases [5]. To further enhance the prediction of progression of CKD various genes and proteins have been identified as molecular biomarker candidates of kidney damage, and their clinical significance has recently been reviewed in detail [6].

High-throughput transcriptomics experiments together with integrative bioinformatics led to the identification of additional novel biomarker candidates. Henger et al. identified a marker set of nine genes (Chemokine (C-C motif) ligand 21, interleukin 8, matrix metalloproteinase 3, 7 and 9, urokinase R, chemokine (C-X-C motif) receptor 5, integrin beta 4, and pleiotrophin), which predicted a progressive course of CKD [7]. Perco et al. showed that histology of zero-hour preimplant biopsies explained only 14% of the variability of one year creatinine whereas a combination of three biomarkers (NLR family, pyrin domain containing 2, immunoglobulin J polypeptide, and the regulator of G-protein signaling 5) without clinical covariables explained 28% [8]. Recently, our group identified activation of intracellular *vascular endothelial growth factor signaling* and *hypoxia response* pathways in microdissected proximal tubule cells from patients with progressive CKD using microarrays and pathway analysis tools. The expression levels of hypoxia-inducible factor-1 alpha and vascular endothelial growth factor-A were significantly superior in predicting clinical outcome as compared to proteinuria, renal function, and degree of histological damage [9].

In the latter two datasets the hyaluronan binding proteoglycan versican (VCAN) – also termed chondroitin sulphate proteoglycan 2 (CSPG2) - showed a significant correlation with progressive decline of post-bioptical renal function in patients with CKD on the one hand and with increased histological damage on the other hand. The aim of the present study was to characterize versican as a novel renal biomarker predicting progressive decline of kidney function in patients with proteinuric nephropathies.

## Results

### Versican is a Marker of Renal Injury and Impaired Renal Function

Data are summarized in table 1. In the histogenomics data set by Perco et al. the expression levels of versican showed significant associations to the degree of acute tubular injury (2.55 fold upregulation), tubular atrophy (2.10 fold upregulation), as well as interstitial fibrosis (1.94 fold upregulation) in zero-hour preimplant renal biopsies [8] (figure S1). Rudnicki et al. analyzed global gene expression in microdissected human proximal tubular epithelial cells isolated from 21 patients with various proteinuric nephropathies. In the array raw data set versican showed a significant mean upregulation by 69% in 3 of 4 versican spots on the arrays when stable (n = 14) and progressive subjects (n = 7) were compared (figure S2) [9]. In kidney biopsies from patients with diabetic nephropathy (DN) versican expression was increased 1.4 fold as compared to patients with minimal change disease (MCD) (p<0.05) (figure S3) [10]. In the 13 diabetic DN biopsies renal versican expression was furthermore significantly correlated with serum creatinine at time of biopsy (spot 1: R = 0.70, p = 0.008, spot 2: R = 0.58, p = 0.04) and with chronic histological damage (spot 1: R = 0.68, p = 0.01, spot 2: R = 0.62, p = 0.03) (figures S4 and S5).

**Table 1:** Microarray data sets used to evaluate the expression of versican in renal pathologies.

Reference	Disease	N	Age (years)	Creatinine (mg/dl)	Tissue	Comparison	Versican upregulation	Significance level ^c^
[8]	Zero-hour transplant biopsies	82	47±16	n.a.	Whole kidney	Acute tubular injury ^b^Tubular atrophy ^b^Interstitial fibrosis ^b^	+2.55 fold+2.10 fold+1.94 fold	P<0.05
[9]	Proteinuric nephropathies ^a^	31	38±15	1.7±1.2	Microdissected PTECs from renal biopsies	Progressive vs stable kidney disease	+1.69 fold	P<0.05 in 3 of 4 versican spots
[10]	LD, CD, MCD, DN	24	47±16	1.4±1.2	Tubulointerstitium fromrenal biopsies	DN vs MCDDN: Pearson correlation with serum creatinineDN: Pearson correlation with histology score	+1.4 foldR = 0.70R = 0.68	P<0.05P<0.05P<0.05

DN diabetic nephropathy, MCD minimal change disease, LD living donors, CD cadaveric donors, PTECs proximal tubular epithelial cells. ^a^ Proteinuric glomerular diseases included focal segmental glomerulosclerosis (FSGS), MCD, IgA nephropathy (IGAN), lupus nephritis (LN), rapid-progressive glomerulonephritis (RPGN), membranoproliferative glomerulonephritis (MPGN), ANCA associated vasculitis (AAV) and hypertensive nephropathy (HTN). Mean values and standard deviations are shown. ^b^ Refer to the text for definition of the histological classes. ^c^ In (8) samples with low and high histological damage were compared and in (9) stable and progressive samples were compared. A two-tailed t-test was performed. In (10) samples from MCD and DN were compared (two tailed t-test), and also a Pearson correlation of creatinine at biopsy and histology score in DN with versican levels was calculated.

However, neither of these studies confirmed the results by real-time PCR or analyzed the expression of the specific versican isoforms V0, V1, V2 and V3 in the respective context covered by each study.

### Renal Versican Expression Correlates with Progressive Decline of Kidney Function

**Figure 1:**
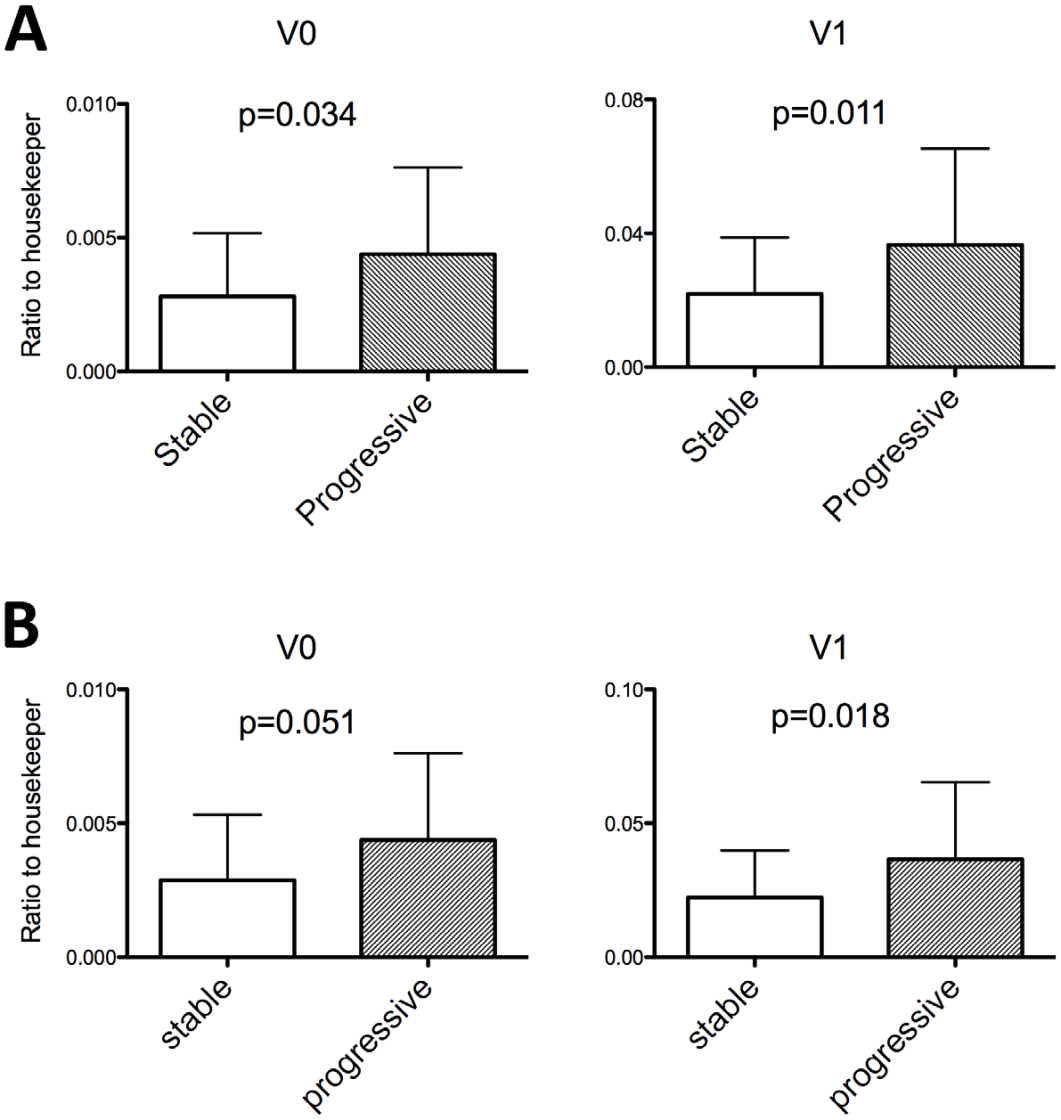
Versican isoform V0 and V1 expression correlates with progression of chronic kidney disease. (A) Whole patient cohort, (B) Patients without ARF. Mean values and standard deviation are shown. p-values of a two-tailed t-test are indicated.

To further characterize renal expression of versican isoforms and the association with clinical parameters we analyzed the expression of versican isoforms V0, V1, V2 and V3 in an independent cohort of 74 patients with various proteinuric kidney diseases (table 2). We did not detect any expression of V2 in these samples, and V3 did not show any correlation with laboratory, clinical or histological parameters (data not shown). No significant correlation of V0 and V1 to age, creatinine, eGFR or proteinuria at time of biopsy was detected, although the correlation of V1 to creatinine at biopsy was of borderline significance (R = 0.220, p = 0.06, table 3). However, both isoforms V0 and V1 did significantly correlate with serum creatinine at time of follow-up (V0 R = 0.337, p = 0.003; V1 R = 0.387, p = 0.001), but not with eGFR or proteinuria. Regarding histological characteristics no significant correlation of V0 or V1 levels with the histological diagnosis, and the degree of tubular atrophy/interstitial fibrosis (TAIF), interstitial inflammation (II), or glomerulosclerosis (GS) was observed. However, V1 levels showed a borderline significant correlation with the degree of TAIF (R = 0.208, p = 0.075) and II (R = 0.199, p = 0.092) (table 3). We classified the set of 74 patients as stable or progressive according to doubling of serum creatinine or reaching of end-stage renal disease (ESRD) during follow-up. As shown in figure 1A the expression of V0 and V1 was higher in progressive disease (V0∶1.56 fold, p = 0.034; V1∶1.67 fold, p = 0.011). Versican V0 and V1 levels did not correlate to eGFR slope (calculated as delta ml/min/1.73 m^2^ per year), neither in the whole group nor when analysis was restricted to subjects with eGFR values <60 ml/min/1.73 m^2^ (data not shown). In the stable cohort 5 patients were identified who had acute renal failure (ARF) at time of biopsy. Since data on renal function of these patients was not available before biopsy, ARF was defined as a decrease of creatinine during follow-up of more than 50%. These 5 patients had a creatinine at time of biopsy of 5.37±1.25 mg/dl and 1.40±0.32 mg/dl after a follow-up time of 55±19 months. When these 5 patients were excluded from the stable cohort, renal function at time of biopsy was significantly different between the stable and the progressive cohort (table 2). V0 and V1 significantly correlated with creatinine at time of biopsy and at time of follow-up, while no significant correlations were found with age and proteinuria. Regarding histological parameters no significant correlations of V0 and V1 with the diagnosis were identified. However, V0 correlated significantly with the degree of GS, while V1 correlated significantly with the degree of TAIF and showed a borderline significant correlation with the degree of II (table 4). As shown in figure 1B the expression of V0 and V1 was still higher in progressive disease (V0∶1.52 fold, p = 0.051; V1∶1.64 fold, p = 0.018) although the level of significance was borderline in the case of V0.

**Table 2:** Patients included in the analysis of versican expression.

Clinical characteristics	All patients [n = 74]	All stable [n = 58]	Stable w/o ARF[n = 53]	Progressive [n = 16]	*p* *	*p* **
Sex (m/f)	49/25	36/22	33/20	13/3	NA	NA
Age (years)	48±16	48±17	48±17	47±14	n.s.	n.s.
Biopsy creatinine (mg/dl)	1.89±1.47	1.76±1.45	1.42±0.88	2.37±1.48	n.s.	0.003
Biopsy proteinuria (g/g)	3.46±3.35	3.42±3.63	3.62±3.72	3.61±2.00	n.s.	n.s.
Follow up time (months)	53±22	55±20	55±20	45±27	n.s.	n.s.
Endpoint: ESRD	12	0	0	12	NA	NA
Endpoint: Doubling creatinine	4	0	0	4	NA	NA
Follow up creatinine (mg/dl)	2.51±2.62	1.34±0.72	1.34±0.74	6.76±2.64	<0.001	<0.001
Follow up proteinuria (g/g)	1.27±1.78	0.65±0.79	0.67±0.80	3.43±2.43	<0.001	<0.001
**Diagnosis**						
DN	3	1	1	2	NA	NA
HTN	7	4	3	3	NA	NA
IGAN	19	13	12	6	NA	NA
MCD	10	10	10	0	NA	NA
FSGS	7	5	5	2	NA	NA
Vasculitis	6	6	5	0	NA	NA
MN	8	5	5	3	NA	NA
IN	4	4	3	0	NA	NA
LN	2	2	2	0	NA	NA
MPGN	2	2	2	0	NA	NA
Goodpasture	2	2	1	0	NA	NA
other	3	3	3	0	NA	NA
RPGN	1	1	1	0	NA	NA

ESRD end-stage renal disease, 2 x creatinine doubling of serum creatinine. Proteinuria was measured as protein/creatinine ratio in g/g. Mean values and standard deviations are shown. Differences between the means were analyzed by a two-tailed t-test. n.s. not significant (p>0.05), NA not applicable. For abbreviation of the histological diagnosis see the *Experimental Procedures* section. ARF Acute Renal Failure. * all stable vs progressive. ** stable w/o ARF vs progressive.

**Table 3:** Clinical and histological correlations of versican isoforms V0 and V1 in the whole CKD cohort.

	V0		V1	
	Pearson R	p	Pearson R	p
**Biopsy**				
Creatinine	0.191	0.103	0.220	0.060
Proteinuria	0.140	0.239	0.044	0.711
Age	−0.008	0.944	−0.040	0.733
**Follow up**				
Creatinine	0.337	0.003	0.387	0.001
Proteinuria	0.079	0.557	0.028	0.833
**Histology**				
Diagnosis	ANOVA	0.163	ANOVA	0.174
Degree of TAIF	0.104	0.378	0.208	0.075
Degree of II	0.131	0.268	0.199	0,092
Degree of GS	0.146	0.222	−0.062	0,605

TAIF tubular atrophy/interstitial fibrosis, II Interstitial inflammation, GS glomerular sclerosis.

**Table 4:** Clinical and histological correlations of versican isoforms V0 and V1 in the CKD cohort excluding patients with ARF.

	V0		V1	
	Pearson R	p	Pearson R	p
**Biopsy**				
Creatinine	0.352	0.003	0.380	0.001
Proteinuria	0.123	0.317	0.026	0.832
Age	-0.023	0.848	−0.054	0.657
**Follow up**				
Creatinine	0.331	0.006	0.381	0.001
Proteinuria	0.067	0.625	0.020	0.903
**Histology**				
Diagnosis	ANOVA	0.080	ANOVA	0.132
Degree of TAIF	0.166	0.180	0.253	0.039
Degree of II	0.169	0.168	0.231	0.058
Degree of GS	0.256	0.036	0.028	0.824

TAIF tubular atrophy/interstitial fibrosis, II Interstitial inflammation, GS glomerular sclerosis.

### Versican Predicts Future Renal Function

Creatinine at time of biopsy (p = 0.002) and the degree of GS (p = 0.006), but neither the degree of proteinuria, nor the extent of TAIF or II were significantly associated with adverse renal outcome. The isoforms V0 and V1 correlated with creatinine at follow-up resulting in R^2^ values of 0.1026 (p = 0.0033) and 0.1421 (p = 0.0006), respectively. The combination of the two parameters GS and creatinine at time of biopsy (model 1) resulted in an adjusted R^2^ of 0.1619 (p = 0.0009). Addition of V0 to this model resulted in an adjusted R^2^ of 0.2012 (p = 0.0004) (model 2). The model with the highest adjusted R^2^ of 0.2438 (p = 0.0001) included the variables GS, creatinine at time of biopsy, and V1 (model 3). The model with both isoforms together with GS and creatinine at time of biopsy (model 4) did not further increase the predictive value (table 5). We also analyzed the prediction of future renal function using the same variables after exclusion of the 5 patients with AKI. In this analysis creatinine at time of biopsy became a strong predictor of creatinine at follow-up with an adjusted R^2^ of 0.34. Although glomerulosclerosis and both versican isoforms also showed significant associations with creatinine at follow-up, the addition of glomerulosclerosis and either V0 or V1 or both isoforms to creatinine did not increase the predictive value of creatinine to a significant extent (table 6).

**Table 5:** Versican isoforms predict the clinical course on top of traditional risk factors – analysis of the whole cohort.

Whole cohort (n = 74)			
	Creatinine at follow-up		
Univariate model	parameter estimate	p-value	adj R^2^
Creatinine at biopsy	0.6372	0.0019	0.1155
Proteinuria at biopsy	0.0249	0.7930	<0
TAIF	0.4871	0.1520	0.0150
II	0.3773	0.2440	0.0053
GS	0.8050	0.0045	0.0983
Versican V0	339.3458	0.0033	0.1026
Versican V1	49.9941	0.0006	0.1421
	**Creatinine at follow-up**		
**Model 1**	**parameter estimate**	**p-value**	**adj R^2^**
Creatinine at biopsy	0.5004	0.0149	
GS	0.5768	0.0429	
total		0.0009	0.1619
	**Creatinine at follow-up**		
**Model 2**	**parameter estimate**	**p-value**	**adj R^2^**
GS	0.5284	0.0579	
Creatinine at biopsy	0.4294	0.0342	
Versican V0	232.2049	0.0411	
total		0.0004	0.2012
	**Creatinine at follow-up**		
**Model 3**	**parameter estimate**	**p-value**	**adj R^2^**
GS	0.6947	0.0119	
Creatinine at biopsy	0.3414	0.0894	
Versican V1	41.2212	0.0052	
total		0.0001	0.2438
	**Creatinine at follow-up**		
**Model 4**	**parameter estimate**	**p-value**	**adj R^2^**
Creatinine at biopsy	0.3364	0.0978	
GS	0.7207	0.0141	
Versican V0	−51.2110	0.7789	
Versican V1	46.5995	0.0552	
total		0.0002	0.2333

For abbreviations see table 3 or 4.

**Table 6:** Versican isoforms predict the clinical course on top of traditional risk factors – analysis of the dataset without ARF patients.

Dataset without ARF patients (n = 69)			
	Creatinine at follow-up		
Univariate model	parameter estimate	p-value	adj R^2^
Creatinine at biopsy	1.4115	<0.001	0.3382
Proteinuria at biopsy	0.0155	0.8752	<0
TAIF	0.6018	0.0962	0.0265
II	0.4850	0.1595	0.0151
GS	0.8082	0.0056	0.0984
Versican V0	332.0354	0.0055	0.0960
Versican V1	48.4368	0.0013	0.1322
	**Creatinine at follow-up**		
**Model 1**	**parameter estimate**	**p-value**	**adj R^2^**
Creatinine at biopsy	1.3211	<0.001	
GS	0.1545	0.5750	
total		<0.001	0.3417
	**Creatinine at follow-up**		
**Model 2**	**parameter estimate**	**p-value**	**adj R^2^**
GS	0.1612	0.5870	
Creatinine at biopsy	1.2331	<0.001	
Versican V0	102.0562	0.9310	
total		<0.001	0.3403
	**Creatinine at follow-up**		
**Model 3**	**parameter estimate**	**p-value**	**adj R^2^**
GS	0.2684	0.3527	
Creatinine at biopsy	1.1344	<0.001	
Versican V1	19.2900	0.1999	
total		<0.001	0.3486
	**Creatinine at follow-up**		
**Model 4**	**parameter estimate**	**p-value**	**adj R^2^**
Creatinine at biopsy	1.1313	<0.001	
GS	0.2790	0.3638	
Versican V0	−19.0025	0.9135	
Versican V1	21.3068	0.3745	
Total		<0.001	0.3382

For abbreviations see table 3 or 4.

### Immunohistochemical Staining Shows Pronounced Versican Expression in the Tubulointerstitial Compartment

**Figure 2:**
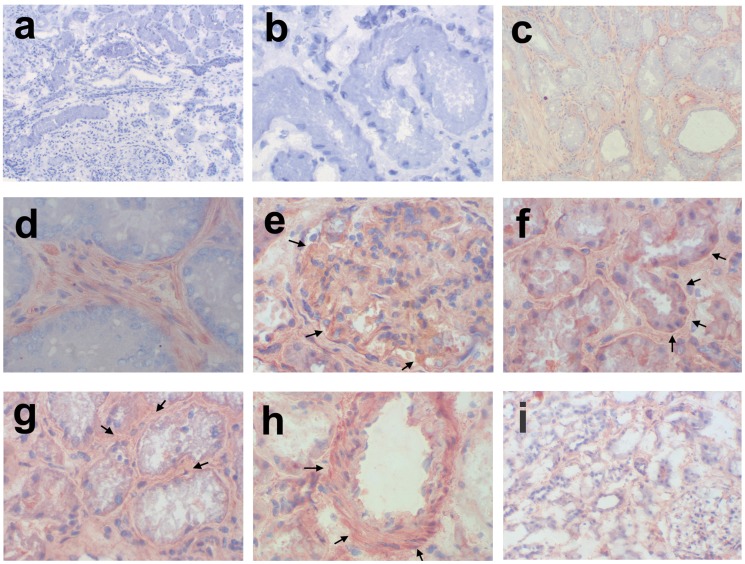
Immunohistochemical staining of versican in positive controls, in human biopsy samples of IgA-Nephropathy (IgAN) and WHO Class IV Lupus Nephritis (LN4) and in healthy kidney tissue. Representative images are shown. a) negative control 100x, b) isotype control 400x, c) and d) positive control in prostate cancer samples (100x and 400x), e) glomerular (LN4, stable), f) tubular (LN4, stable), g) interstitial (IgAN, stable), h) vascular (IgAN, stable) expression (all 400x) of versican in human biopsy samples of renal disease, and i) healthy kidney tissue from a tumor nephrectomy (100x).

Next we evaluated the potential origin of versican expression by immunohistochemistry in a healthy control and in representative biopsies from patients with proteinuric nephropathies. We found ubiquitous versican protein expression in the glomerular and in the tubulointerstitial compartment as well as in blood vessels (figure 2). Glomerular expression was rather weak, and the tubular expression was more pronounced at the basolateral membrane. In areas of marked interstitial fibrosis versican expression was also increased showing a fibrillar pattern. In renal blood vessels no expression of versican was detected in the intima, but there was a strong signal in the media and in the adventitia. In healthy renal tissue versican expression was generally lower than in the diseased cases. A weak signal was seen mainly in the tubulointerstitium.

### Versican is Expressed in Renal Epithelial Cells and in Fibroblasts in vitro

**Figure 3:**
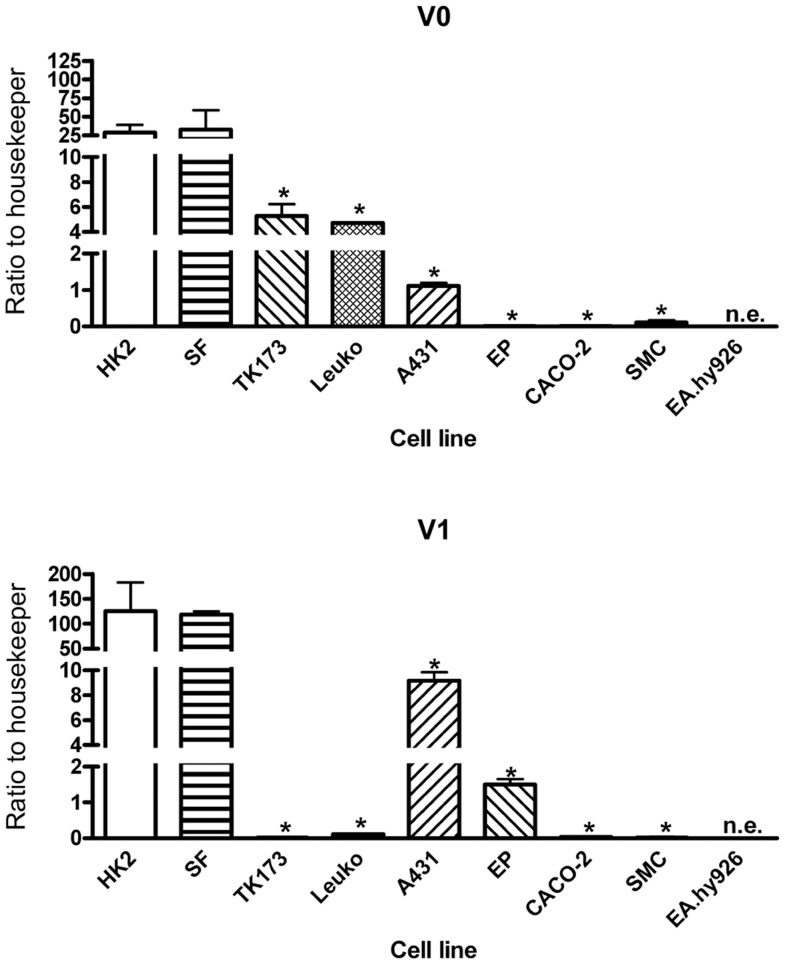
Expression of versican mRNA in vitro. Versican is expressed in various renal and non-renal cell lines. HK2 renal proximal tubule cells, SF primary skin fibroblasts, TK173 kidney fibroblasts, A431 epidermoid carcinoma cell line, EP prostate epithelial cells, CACO2 colon carcinoma cells, CMS primary smooth muscle cells, EA.hy926 endothelial cells. The expression values are shown as ratio to PPIA. The mean and the standard deviation from n = 3 experiments is shown. * p<0.05 as compared to HK2 cells measured by a two-tailed t-test. n.e. not expressed.

We further analyzed cell and organ specificity of versican expression. Human proximal tubule cell line HK2 and human skin fibroblast (SF) cell line showed highest expression of V0 and V1 (figure 3). Apart from this relatively similar expression pattern in these three cell lines, we also detected cell type specific differences in expression between the isoforms V0 and V1. Human kidney fibroblasts TK-173, leukocytes, and the epithelial cell line A431 showed a V0 expression which was approximately 5 to 25 times less than in HK2 cells. The expression of V0 was extremely low in prostate epithelial cells, colon epithelial cells, smooth muscle cells and also in control (“healthy”) kidney tissue (0.06% to 0.3% as compared to HK2). The results were similar for V1 (0.01% to 7% as compared to HK2), with the exception that V1 expression was also very low in kidney fibroblasts and leukocytes (0.01% to 0.08% as compared to HK2). We did not detect any versican expression in endothelial cells.

### TGF-ß1 Upregulates Versican in Fibroblasts

**Figure 4:**
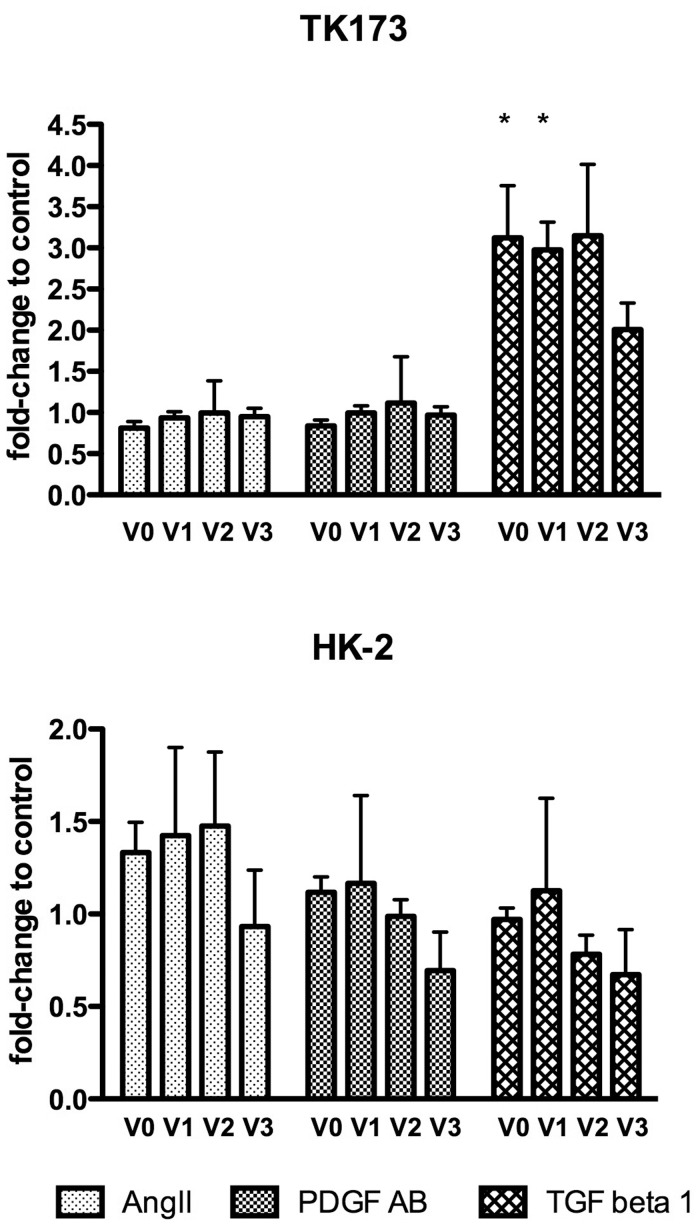
Versican regulation in fibroblasts and tubule cells. TK-173 and HK-2 cells were treated with Ang II, PDGF-AB and TGF-ß1 for 24 hours. After RNA isolation all 4 versican isoforms were analyzed by real-time PCR (n = 3 assays per ligand). Results shown are mean ratios to the housekeeper gene PPIA ± standard deviation. *p<0.05 as compared to controls.

Based on the high basal expression of versican in tubule cells and in fibroblasts we next examined the effect of angiotensin (Ang) II, platelet-derived growth factor (PDGF)-AB and transforming growth factor (TGF) beta-1 on the expression of all versican isoforms in HK-2 and TK-173 cells. We selected these ligands because of their known regulatory effect on versican expression in smooth-muscle cells, and their well-established profibrotic and proinflammatory characteristics. When HK-2 and TK-173 cells were treated with Ang II or PDGF-AB, we did not detect any regulation of any of the versican isoforms. However, treatment with TGF beta-1 resulted in a significant upregulation of V0 and V1 by approx. 3.5 fold in the fibroblast cell line TK-173. We did not detect any significant effect of TGF-ß1 on versican isoform expression in the proximal tubule cell line HK-2 (figure 4).

### Versican is Upregulated in Rodent Models of Kidney Diseases

**Figure 5:**
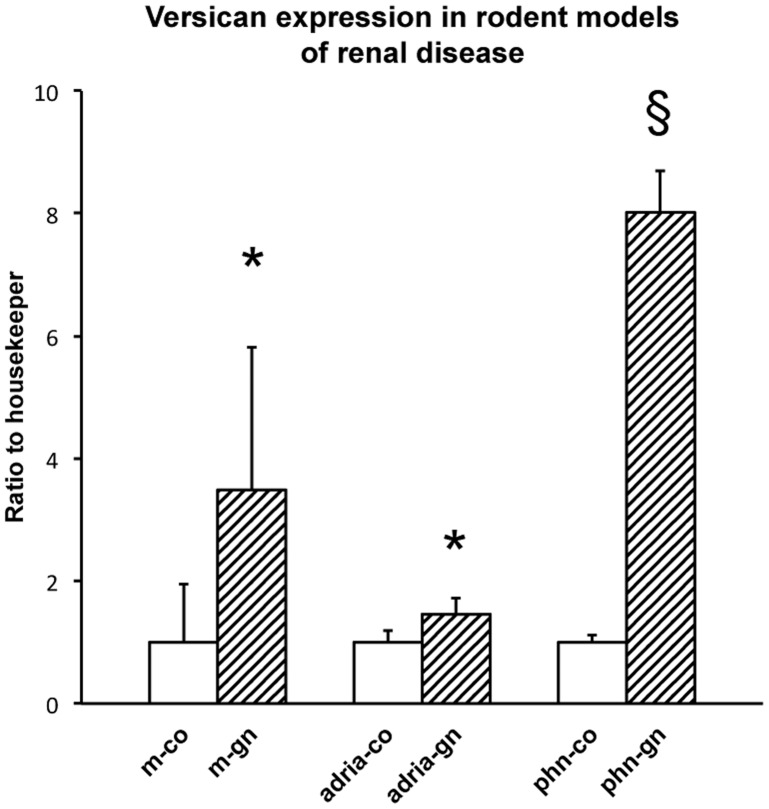
Versican expression in rodent models of renal disease. Versican is significantly upregulated in various mouse and rat models of glomerulonephritis. Mean values and standard deviations are shown. Two-tailed t-test was performed. * p<0.05, § p<0.001, co controls, gn glomerulonephritis, m mouse, phn Passive Heyman Nephritis, adria Adriamycin rats.

**Figure 6:**
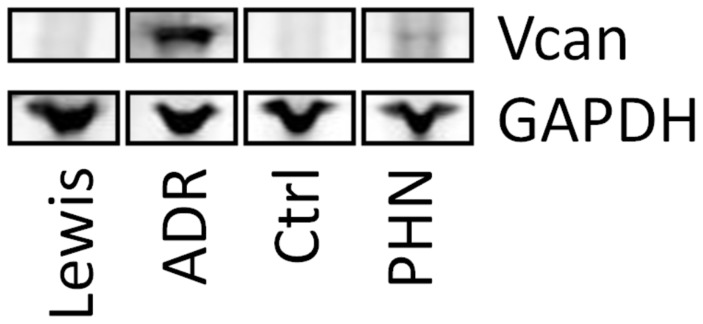
Expression of versican protein in vivo. Versican protein expression is induced in two rat models of glomerulonephritis (Adriamycin-induced glomerulonephritis (ADR) and Passive Heymann Nephritis (PHN)) compared to respective controls, Lewis and Ctrl. Versican expression was analyzed in 6 samples of each group. Figure 6 shows one representative sample each.

We determined the relevance of our findings by analyzing renal versican mRNA expression in mouse and rat models resembling human glomerular pathologies. The expression values in corresponding controls were arbitrarily set to 1. Versican was upregulated 3.5 fold (p = 0.02) in mice with accelerated nephrotoxic serum nephritis, a model of proliferative glomerulonephritis, after 14 days (figure 5). In rats with adriamycin-induced nephropathy, that mimics aspects of human MCD and focal-segmental glomerulosclerosis (FSGS), a 1.5 fold (p = 0.03) increase in versican mRNA as compared to controls was found at 21 days. Finally, a highly significant upregulation of renal versican, i.e. 8.0 fold (p<0.001) increase over control, was observed in rats with Passive Heymann Nephritis (PHN) - a severe model of proteinuric nephropathy which partly resembles human membranous nephropathy (MN) - studied at 6 months. In ADR and in PHN nephropathy versican expression was also increased on the protein level (figure 6).

## Discussion

In this work we describe the two versican isoforms V0 and V1 as novel biomarker candidates with the potential to predict progression of CKD already at an early stage.

Versican is an extracellular matrix (ECM) protein belonging to the family of hyaluronan-binding proteoglycans showing expression in a variety of connective tissues. Four splice variants of human versican have been identified (V0, V1, V2 and V3), which result from alternative splicing of the two central exons 7 and 8 encoding the central glycosaminoglycan carrying regions, glycosaminoglycan alpha (GAG-α) and beta (GAG-β) [11]. It is V0 and V1, which are the predominant versican isoforms in stroma tissues of most cancers [12], [13], [14], while V2 expression seems to be restricted to the central nervous tissue [15]. V3 does not contain any GAG attachment sites and has been proposed to act as a regulatory protein [16]. Versican is involved in cell proliferation, cell adhesion, migration, and extracellular matrix assembly [17]. The expression of versican in smooth muscle cells is induced by various mitogens such as transforming growth factor beta-1, platelet derived growth factor, epidermal growth factor, basal fibroblast growth factor, and interleukin 1 beta via phosphatidylinositol 3-kinase–protein kinase B signaling, and is regulated by downstream transcription factors such as cAMP-responsive element binding protein, nuclear factor-kB (NF-kB), and p53 [18].

We identified versican as a marker of histological damage in zero-hour biopsies, of progressive decline of renal function in CKD and in DN in publicly available transcriptomics data sets. Furthermore, versican V0 and V1 levels significantly increased the predictive value of conventional clinical and histological risk factors for renal disease progression in an independent cohort of patients with proteinuric renal diseases. In the field of cancer research several authors have previously identified high levels of tissue versican expression as an indicator for poor outcome in malignant diseases such as prostate cancer [19] and breast cancer [12]. Data on the association of versican expression with clinical outcome in kidney disease are scarce. In a genomics analysis in ageing kidneys, Melk et al. found renal versican RNA levels to be increased 2.90 fold in kidney tissue from older patients as compared to samples from adults. The highest differential versican expression (3.64 fold) was observed between samples with higher and lower degree of histological damage, thus also indicating a correlation with histological damage [20]. In kidney transplantation Einecke et al. evaluated global gene expression in 105 renal biopsies [21]. Versican was one of the most significant genes, which predicted renal graft loss with a 3.42 times higher expression in failed grafts as compared to censored grafts. Interestingly, in our independent cohort of 74 patients with various renal histologies versican expression levels did not correlate with the degree of TAIF, II or GS, although the correlation of V0 with glomerulosclerosis and of V1 with TAIF and II reached significant levels after exclusion of patients with ARF (see below). This association of versican with histological injury is in line with findings from several authors where (not isoform specific) versican expression was increased in renal tissue with high degree of histological injury [8], [10], [20]. This association is further supported by the borderline significant correlation of versican levels with serum creatinine at time of biopsy (which became significant after exclusion of ARF patients) in our cohort and in the findings from Schmid et al. [10]. Therefore, we cannot rule out the possibility that the predictive characteristics of versican V0 and V1 expression values on progression of renal failure are not – at least in part – due to increased damage of kidney tissue and poor kidney function already at time of biopsy.

Versican levels did neither correlate with proteinuria nor did proteinuria predict adverse renal outcome. This lack of association of chronic histological changes and of proteinuria with prognosis might point to a selection bias in which patients with an acute form of glomerulonephritis and not a chronic presentation undergo kidney biopsy, thus limiting the generalizability of these results. To address this issue we identified 5 patients with ARF at time of biopsy in the stable cohort. Although these patients showed lower renal versican V0 and V1 expression than other stable patients as well as the progressive cohort, the differences did not reach statistical significance (data not shown). After exclusion of these 5 patients V0 and V1 levels were still higher in progressive subjects (V0 p = 0.051, V1 p = 0.018). Furthermore, V0 and V1 showed positive correlations to creatinine at time of biopsy and to the degree of histological damage. However, then the predictive value of creatinine at time of biopsy increased substantially (R^2^ = 0.34), and was not improved by the degree of glomerulosclerosis or V0 or V1 levels to a significant extent. These results in patients without ARF are in line with published data, which show that basal renal function is a robust marker for prediction of progression of CKD. However, in the clinical setting of deteriorated renal function at time of biopsy it is often difficult to predict future renal function. In this setting renal versican expression might serve as a potential candidate to predict worse renal outcome.

Results in humans were reproduced in three animal models of nephropathies in which renal versican expression was significantly increased in diseased animals as compared to controls. Analysis of versican RNA expression *in vitro* showed significantly higher expression levels of V0 and V1 in renal tubule cells and in fibroblasts than in other cell lines. These findings were corroborated in biopsies from patients with IgA nephropathy (IgAN) and Lupus nephritis showing versican protein expression mainly in tubule cells, the ECM and interstitial fibroblasts. Bode-Lesniewska et al. showed that the V0 and V1 isoforms of versican are expressed in various tissues and epithelial cells, but not in renal tubules and glomeruli [22]. However, versican was detected in the surrounding interstitial tissue and in sclerosed glomeruli. These contradicting results could be explained by the fact that renal tubular expression of versican might only be activated in the diseased state. This hypothesis is supported by our findings of very low versican expression in control kidney tissue. Similar to our results a higher expression of versican in the fibromuscular stroma was reported in prostate cancer tissue [19] and in the stroma of nodules in benign prostatic hyperplasia [23] but not in prostate epithelial cells when compared with healthy prostate samples. These studies suggest that versican is mainly expressed in fibroblasts, and also deposited as part of the ECM, respectively.

We detected an induction of versican expression in fibroblasts by TGF beta-1. It has been proposed that versican might be involved in epithelial-mesenchymal-transition (EMT) and mesenchymal-epithelial-transition (MET). In NIH3T3 fibroblasts V1 but not V2 induced MET with a switch in cadherin expression from N- to E-cadherin, reduced expression of vimentin, and increased expression of occludin resulting in a polarized and epithelial phenotype [24]. Furthermore, V1 enhanced cell proliferation, modulated cell cycle progression, and inhibited apoptosis via the activation of epithelial growth factor receptor expression, modulation of its downstream pathways and by inducing degradation of the cyclin-dependent kinase inhibitor p27 [25]. In the context of progressive renal disease these observations would suggest “protective” characteristics of at least versican isoform V1, in which overexpression in fibroblasts induces an epithelial phenotype and enhances gap junction communications.

On the other hand it was shown that versican binds to adhesion molecules on the surface of leukocytes such as P- and L-selectins, and also CD44 [26]. Furthermore, the chondroitin sulphate chains of versican mediate the binding of several chemokines involved in the recruitment of mononuclear leukocytes [27]. Together with versicańs ability to bind to ECM components [28] increased versican levels might hence sustain an inflammatory microenvironment, thus promoting fibrogenesis and progression of CKD.

In conclusion, we identified renal expression of versican isoforms V0 and V1 as markers for unfavourable renal prognosis. Due to the highly interactive characteristics of versican this molecule represents a promising candidate for further studies of renal pathologies. However, before versican levels can be used as a prognostic marker candidate these findings have to be replicated in an independent cohort of well characterized patients with sufficient statistical power.

## Materials and Methods

### Ethics Statement

Before performing a routine kidney biopsy due to a clinical indication a written informed consent is obtained from every patient. Usually two biopsy cores are retrieved, one for routine histological analysis, which is fixed immediately with formalin and embedded in paraffin, and one for immunofluorescence analysis, which is embedded in Tissue-Tek®OCT™ (Sakura, Alphen aan den Rijn, the Netherlands) compound and processed as cryocut sections. After the diagnostic workup is finished the surplus cryocut sections are stored at −80°C. The Institutional Review Board of the Medical University of Innsbruck has accredited the use of surplus material from routine kidney biopsies for research purposes.

### Microarray Data Sets on Versican Expression in Kidney Tissue

We extracted versican RNA expression values from three transcriptomics studies in the context of kidney transplantation [8], chronic kidney disease [9] and diabetic nephropathy [10]. Versican levels were investigated in the context of the clinical questions of the respective papers (table 1). Raw data from the array experiments and supplemental figures are accessible via www.microarray.at.

### Patients and Kidney Biopsies of the Validation Cohort

We used kidney biopsies obtained from 74 patients with proteinuric renal diseases during their routine diagnostic workup. Diagnoses included focal segmental glomerulosclerosis (FSGS), minimal change disease (MCD), IgA nephropathy (IGAN), lupus nephritis (LN), rapid-progressive glomerulonephritis (RPGN), membranoproliferative glomerulonephritis (MPGN), ANCA associated vasculitis (AAV), hypertensive nephropathy (HTN), membranous nephropathy (MN), Goodpasture syndrome, interstitial nephritis (IN) and other. Clinical follow-up data is summarized in table 2. Progressive disease was defined as doubling of serum creatinine during follow-up or end-stage renal disease. All other patients were defined as stable. An independent pathologist scored the degree of tubular atrophy/interstitial fibrosis (TAIF), glomerular sclerosis (GS) and interstitial inflammation (II) following a semiquantitative grading system on haematoxylin/eosin and periodic-acid-Schiff- or Pearse-stained sections.

### Correlation of Versican Isoform mRNA Levels with Clinical Parameters and Outcome

Correlations of versican isoforms V0 and V1 were calculated to (i) clinical parameters at time of biopsy (age, creatinine, eGFR-MDRD, proteinuria), (ii) clinical parameters at follow-up (creatinine, eGFR-MDRD, proteinuria), and (iii) histological parameters (diagnosis, degree of TAIF, GS and II). The Pearson correlation coefficient was used for all analyses except for the correlation of versican levels with histological diagnosis for which an ANOVA test was used. We determined the strength of versican isoforms V0 and V1 along with traditional risk factors for progression such as creatinine at time of biopsy, proteinuria, degree of TAIF, GS and II to predict patient outcome. Creatinine at follow-up was used as outcome variable in the linear regression models.

### RNA Isolation and Real-time PCR

RNA was isolated using the RNeasy® Micro Kit (Qiagen, Hilden, Germany). RNA was reverse transcribed into cDNA with the High Capacity cDNA reverse Transcription kit (Applied Biosystems, Carlsbad, CA). Preamplification was performed using TaqMan® Gene Expression Assays (*vide infra*) and the TaqMan® PreAmp Master Mix. The preamplified cDNA was analyzed in duplicates on the 7500 Fast Real-Time PCR System (Applied Biosystems, Carlsbad, CA) using the following inventoried TaqMan® Gene Expression Assays: PPIA (cyclophilin A; Hs99999904_m1), VCAN0 (Hs01007944_m1), VCAN1 (Hs01007937_m1), VCAN2 (Hs01007943_m1) and VCAN3 (Hs01007941_m1). The relative amounts of transcripts for each gene were normalized to the reference gene cyclophilin A (PPIA).

### Cell Culture

Human renal proximal tubule cells (HK2), colon carcinoma cells (CACO2), epidermoid carcinoma cells (A431), and human endothelial cells (EA.hy926) were purchased from the American Type Culture Collection. The human kidney fibroblast cell line TK-173 was provided by Gerhard A. Müller, Göttingen, Germany [29]. Immortalized prostate epithelial cells (EP156T, EP153T) [30], primary smooth muscle cells (SMC) [31] and primary skin fibroblasts (SF) [32] were obtained from Helmut Klocker at the Department of Experimental Urology from the Medical University Innsbruck. Leukocyte cDNA was obtained from the Human MTC™ Panel I and Panel II (Clontech, Mountain View, CA). Kidney tissue was obtained from the unaffected part of a kidney, which was nephrectomized due to renal cell carcinoma. HK-2 cells were cultured in keratinocyte-serum-free medium (KSFM) containing 10% FCS, 5 ng/ml recombinant epidermal growth factor (rEGF), and 0.05 mg/ml bovine pituitary extract (BPE) and 1% PenStrep. TK173 fibroblasts were cultured in DMEM containing 10% FCS, 1% Glutamax (Life Technologies Corporation, Carlsbad, CA) and 1% PenStrep. After 24 h in serum-free medium HK-2 and TK173 cells were cultured for 24 h in the presence of angiotensin (Ang) II (10^−7^ mM), platelet-derived growth factor (PDGF)-AB (10 ng/ml) or transforming growth factor (TGF) beta-1 (10 ng/ml). RNA isolation and quantitative real-time PCR of the versican isoforms was performed as described above, but RNA was not pre-amplified.

### Versican Immunohistochemistry

Frozen sections from six patients (3 IgAN, 3 class IV lupus nephritis) were stained for human versican protein. Two patients (both IgAN) showed severe renal function impairment and a progressive course of disease, while the other 4 showed conserved renal function and a stable course of disease. As healthy control tissue we used an unaffected part of a tumor nephrectomy specimen. The sections were fixed in cold acetone. The sections were then incubated at 4°C overnight with a 1∶400 dilution of the primary antibody (rabbit anti-human Versican, sc-25831, Santa Cruz, CA). Versican was detected by the Vectastain Elite ABC Kit (Vector Laboratories, Burlingame, CA). This system uses a biotin-conjugated secondary antibody (1∶1000), avidin and biotinylated horseradish peroxidase, and 3-amino-9-ethylcarbazole (AEC) as the chromogen for visualization. All sections were counterstained with hematoxylin.

### Animal Models of Renal Disease

We investigated versican expression in 3 rodent models of renal disease: (A) Accelerated anti-glomerular basement membrane nephritis was induced in n = 8 mice as described previously [33], and n = 8 untreated animals served as controls. In brief, 8- to 12-wk-old male C57Bl/6J mice were obtained from Charles River (Sulzfeld, Germany). The animals were preimmunized subcutaneously with 2 mg/ml rabbit IgG (Jackson Immuno Research Laboratories, West Grove, PA) dissolved in incomplete Freund's adjuvant (Sigma, St. Louis, MI) and nonviable desiccated *Mycobacterium tuberculosis* H37a (Difco Laboratories, Detroit, MI). After 5 days, heat-inactivated rabbit anti-mouse GBM antiserum was injected *via* the tail vein. The animals were sacrificed after 14 days. Austrian veterinary authorities approved these animal experiments (GZ 66.011/0.111–11/10b/2008; Bundesministerium für Wissenschaft und Forschung). (B) Passive Heymann Nephritis (PHN) was induced in n = 5 2-month old male Sprague-Dawley rats (Charles River Italia s.p.a., Calco, Italy) by a single intravenous injection of 0.4 ml/100 g body weight of rabbit anti-Fx1A antibody, followed 7 days later by unilateral nephrectomy to accelerate the onset of renal damage [34]. Animals were sacrificed after 6 months. Age-matched Sprague–Dawley rats (n = 5) were used as control. (C) Adriamycin nephropathy was induced in n = 5 male Lewis rats (Charles River Italia s.p.a., Calco, Italy) by a single intravenous injection of ADR (Adriblastina, Pfizer Italia s.r.l, Latina, Italy) at the dose of 5 mg/kg [35]. Five Lewis rats intravenously injected with saline served as controls. Animals were sacrificed after 21 days. Animal care and treatment were in accordance with current law [36]. Animal studies were submitted to and approved by the Institutional Animal Care and Use Committee of “Mario Negri” Institute, Milan, Italy. Renal expression of versican was analyzed by real-time PCR following the above outlined protocol using the inventoried TaqMan Gene Expression Assays for mouse versican (Mm01283063_m1), mouse 18s ribosomal RNA (Mm03928990_g1), rat versican (Rn01493755_m1) and rat cyclophilin A (Rn00690933_m1). The TaqMan Gene Expression Assays for mouse-versican and rat-versican used in this study are not isoform-specific but rather allow the detection of the expression of any of the known versican isoforms.

### Western Blot

The protocol has been previously published [37]. In brief, 1×1×2 mm blocks of rat kidney tissue were lysed in 250 µl of SDS sample buffer (NuSep Inc., Bogart, GA). 15 µl per lane were resolved using 4 to 12% Bis-Tris gels (Invitrogen, Leek, The Netherlands) and transferred onto a nitrocellulose membrane (Invitrogen). Primary antibody (rabbit anti-human versican, sc-25832, Santa Cruz Biotechnology Inc., Santa Cruz, CA) was incubated at 4°C over night. After washing, membranes were incubated with fluorescence-labeled secondary antibodies (Molecular Probes, Eugene, OR) and subsequently scanned using the Odyssey infrared imaging system (LiCor Biosciences, Lincoln, NE).

## Supporting Information

Figure S1 Versican expression in zero-hour preimplant renal biopsies. Data were extracted from the publication by Perco et al. Increased versican expression is found in biopsies with a higher level of histological damage, such as glomerulosclerosis (gs), arteriolosclerosis (as), interstitial fibrosis (if), interstitial inflammation (ii), tubular atrophy (ta) and acute tubular injury (ati). The degree of histological damage was assessed using a semiquantitative grading system: 0 - no; 1 - minor; 2 - moderate; 3– severe. Samples with grade 0 were defined as “low” in gs, as, if, ii and ta, while samples with grades 1–3 were defined as “high”. In the case of ati samples with grade 0–1 were defined “low” and samples with grades above 2 were defined as “high”. * depicts significant differences.
(JPG)

Figure S2 Versican expression in microdissected tubule cells from human subjects with stable and progressive course of CKD. Data extracted from microarray raw data from our group [9]. The accession numbers represent four different versican spots on the arrays. Three of those four showed significant differences in expression. * p<0.05.
(JPG)

Figure S3 Versican expression in living donors, minimal change disease, cadaveric donors and in diabetic nephropathy. Data extracted from the raw data provided by Schmid et al [10]. NM_004385 and BF218922 represent two different spots on the respective microarrays. * p<0.05
(JPG)

Figure S4 Versican expression and serum creatinine at time of biopsy in diabetic nephropathy. Data extracted from the raw data provided by Schmid et al [10].
(JPG)

Figure S5 Versican expression and histology score in diabetic nephropathy. Data extracted from the raw data provided by Schmid et al [10].
(JPG)
