# Supplementary figures and images for: Increased Renal Versican Expression Is Associated with Progression of Chronic Kidney Disease
Source: PLoS One. 2012 Sep 14;7(9):e44891. doi: 10.1371/journal.pone.0044891 (PMC3443107; doi:10.1371/journal.pone.0044891)

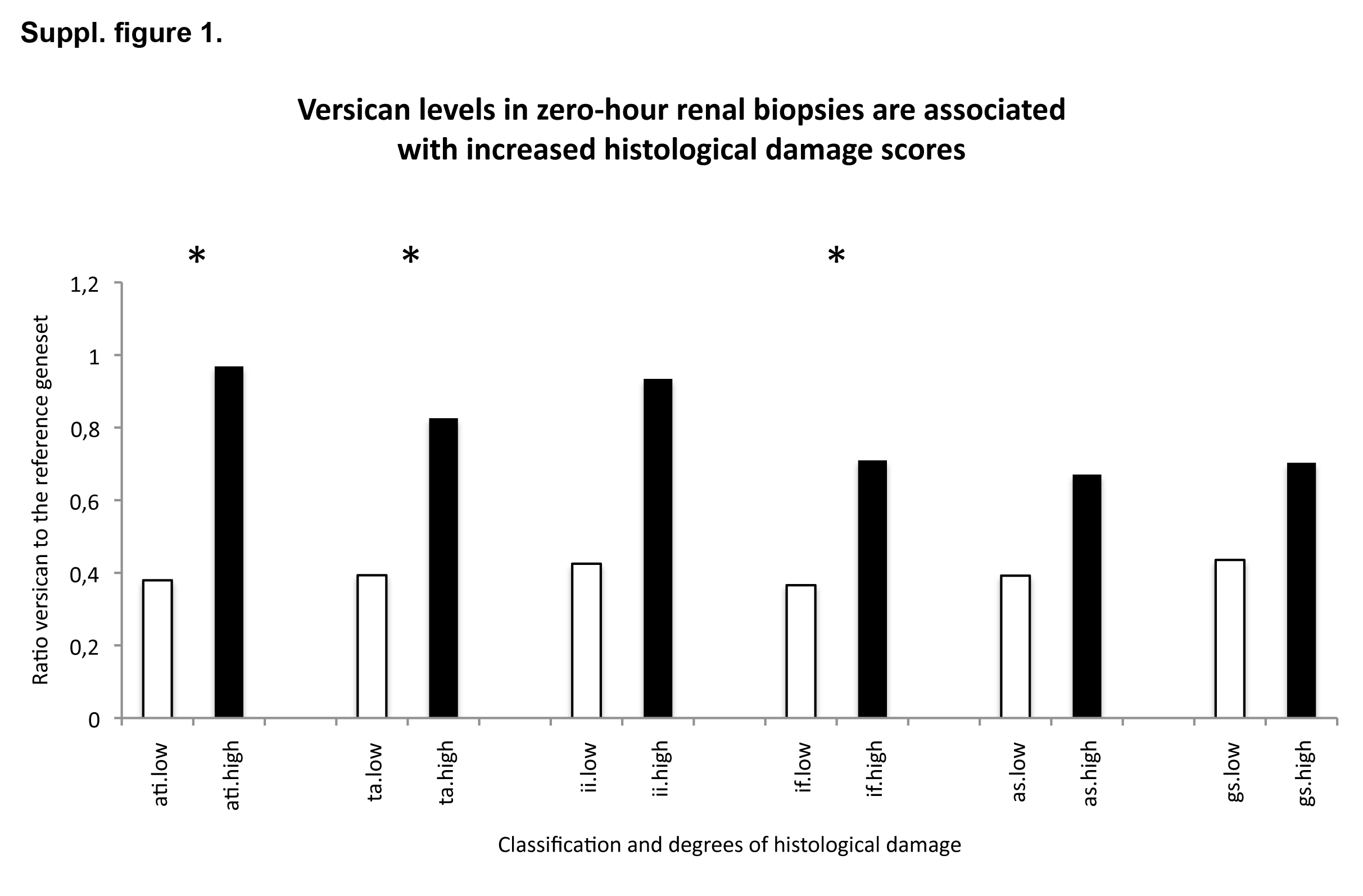

Supplement: Figure S1 — Versican expression in zero-hour preimplant renal biopsies. Data were extracted from the publication by Perco et al. Increased versican expression is found in biopsies with a higher level of histological damage, such as glomerulosclerosis (gs), arteriolosclerosis (as), interstitial fibrosis (if), interstitial inflammation (ii), tubular atrophy (ta) and acute tubular injury (ati). The degree of histological damage was assessed using a semiquantitative grading system: 0 - no; 1 - minor; 2 - moderate; 3– severe. Samples with grade 0 were defined as “low” in gs, as, if, ii and ta, while samples with grades 1–3 were defined as “high”. In the case of ati samples with grade 0–1 were defined “low” and samples with grades above 2 were defined as “high”. * depicts significant differences. (JPG) [file pone.0044891.s001.jpg]

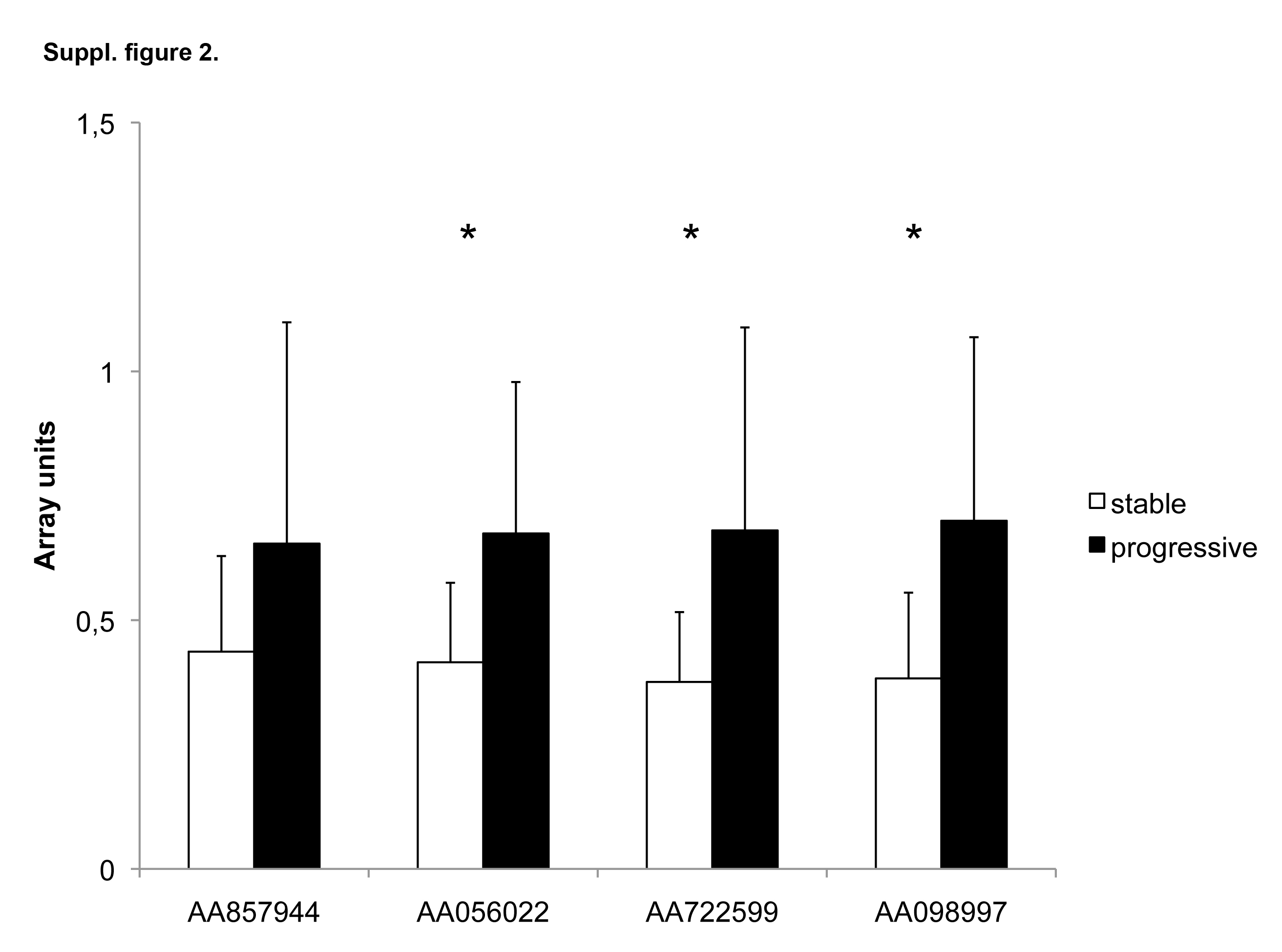

Supplement: Figure S2 — Versican expression in microdissected tubule cells from human subjects with stable and progressive course of CKD. Data extracted from microarray raw data from our group [9]. The accession numbers represent four different versican spots on the arrays. Three of those four showed significant differences in expression. * p<0.05. (JPG) [file pone.0044891.s002.jpg]

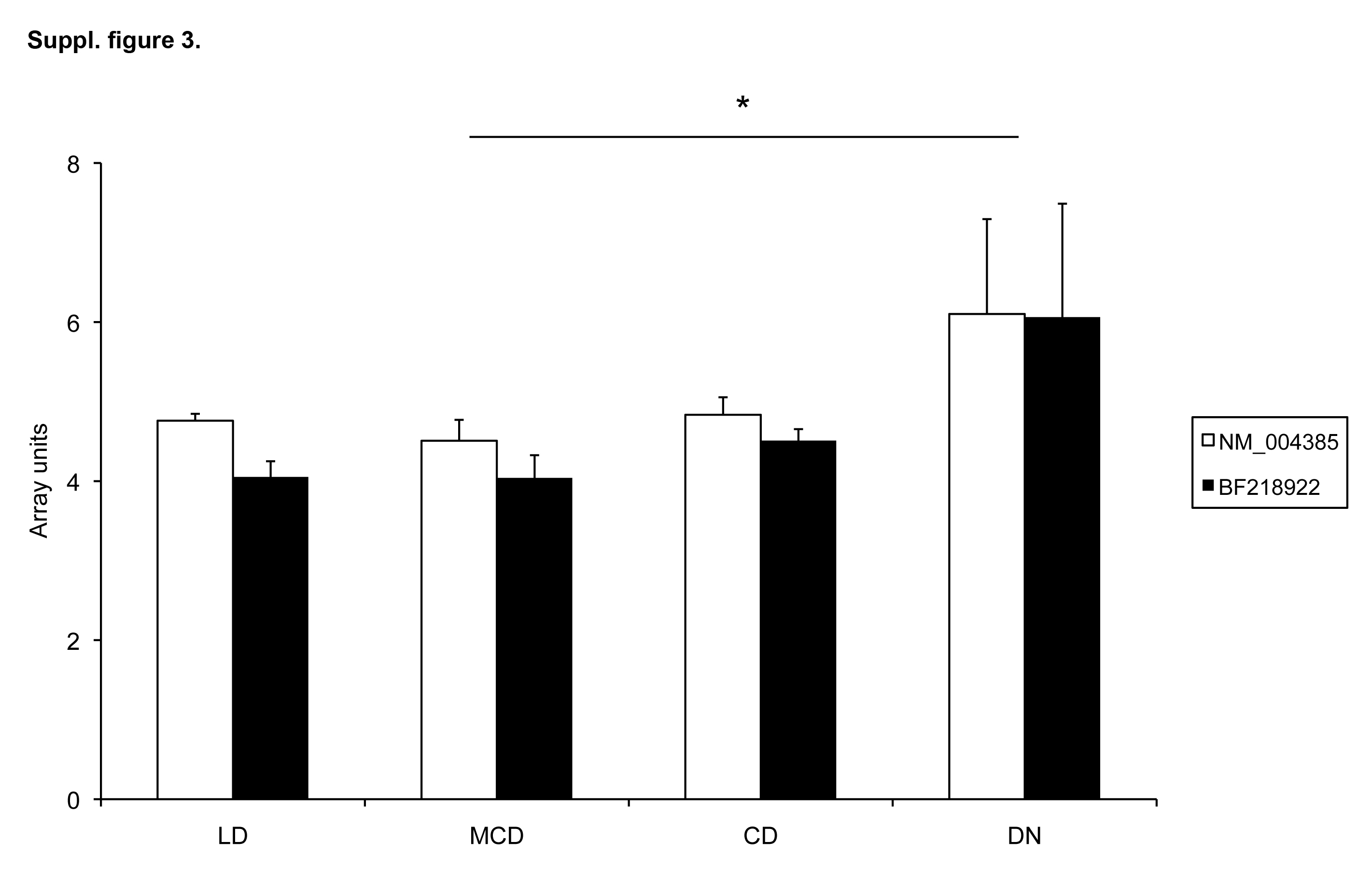

Supplement: Figure S3 — Versican expression in living donors, minimal change disease, cadaveric donors and in diabetic nephropathy. Data extracted from the raw data provided by Schmid et al [10]. NM_004385 and BF218922 represent two different spots on the respective microarrays. * p<0.05 (JPG) [file pone.0044891.s003.jpg]

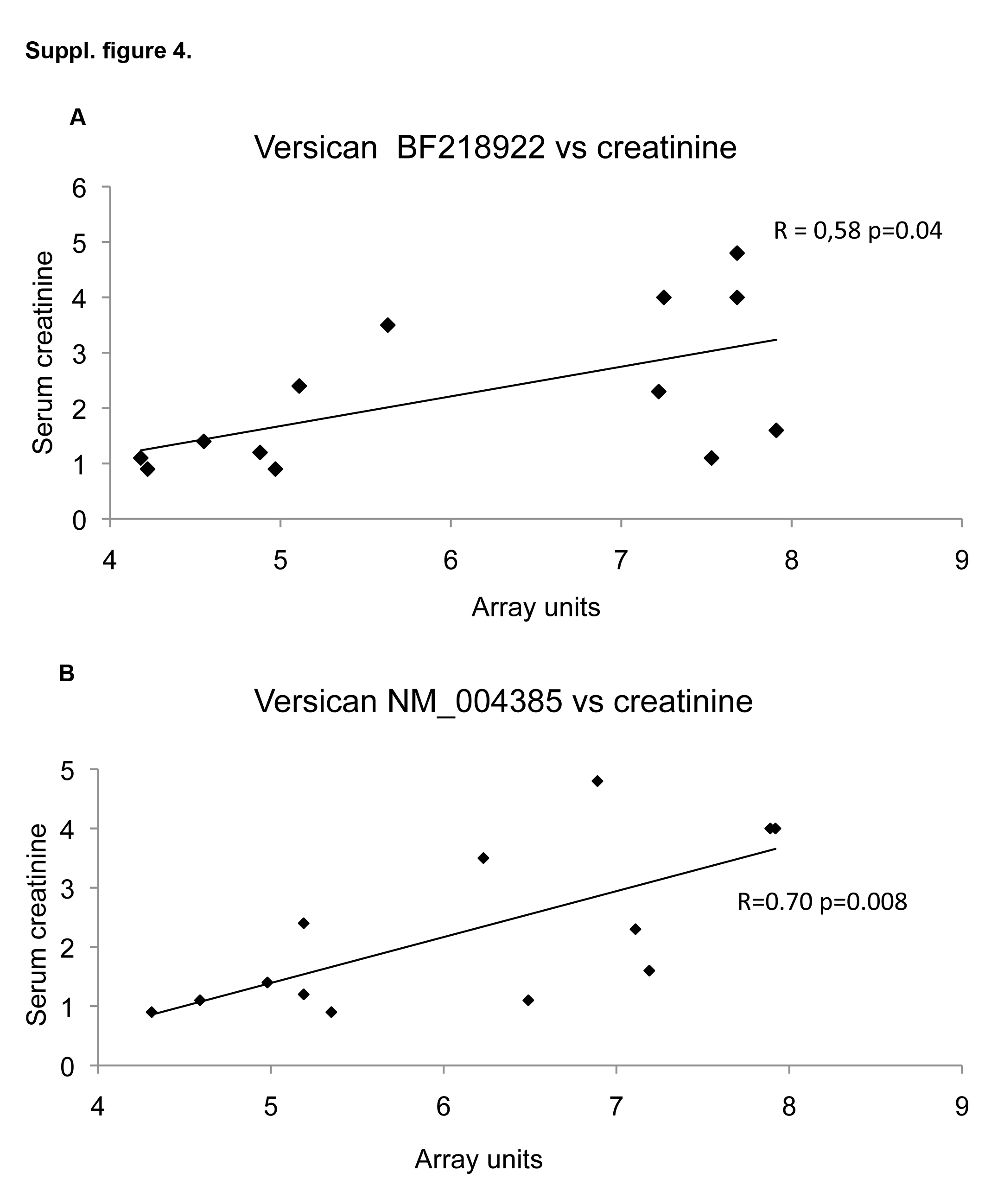

Supplement: Figure S4 — Versican expression and serum creatinine at time of biopsy in diabetic nephropathy. Data extracted from the raw data provided by Schmid et al [10]. (JPG) [file pone.0044891.s004.jpg]

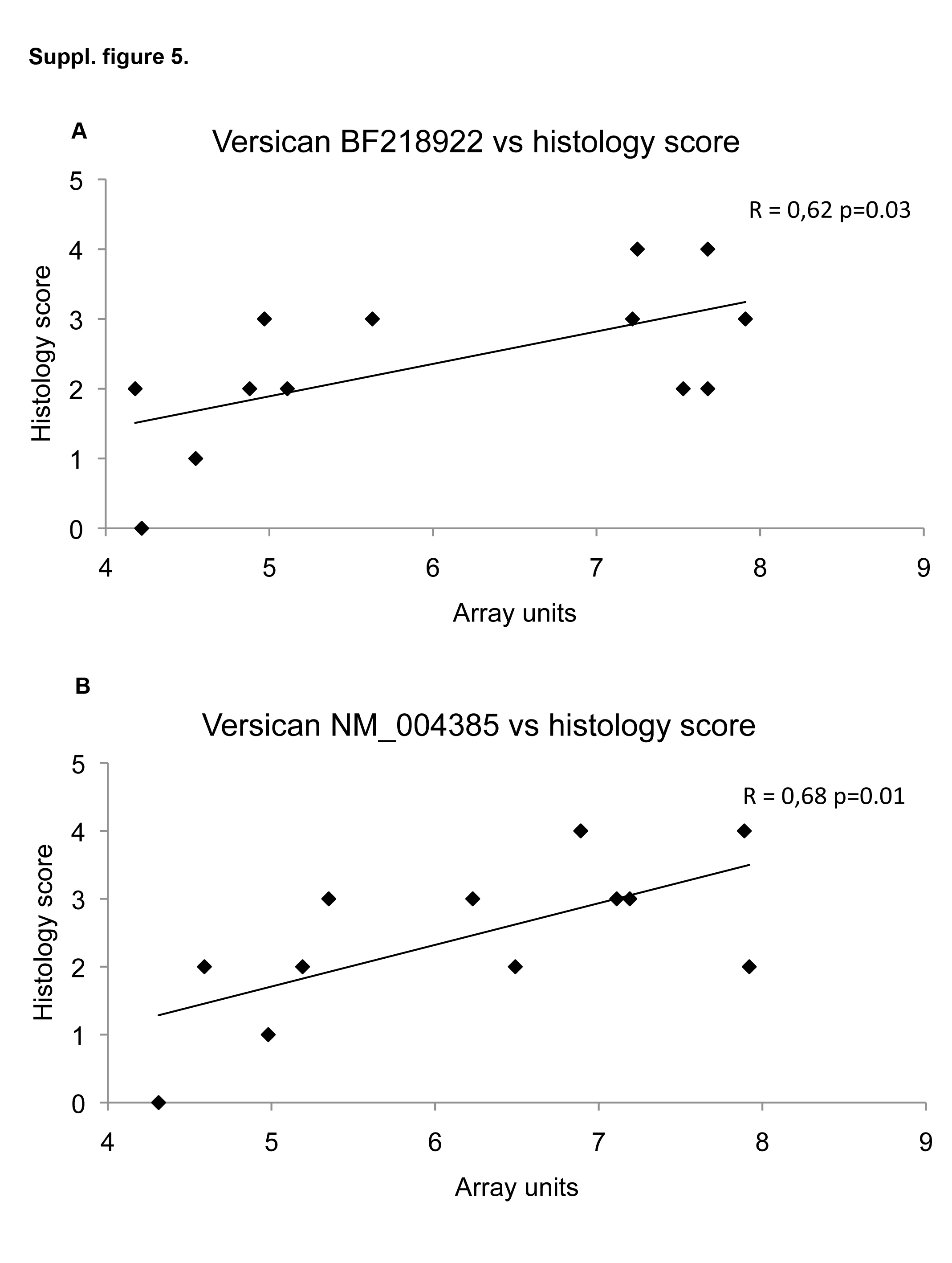

Supplement: Figure S5 — Versican expression and histology score in diabetic nephropathy. Data extracted from the raw data provided by Schmid et al [10]. (JPG) [file pone.0044891.s005.jpg]
